# Spatial density estimates of Eurasian lynx (*Lynx lynx*) in the French Jura and Vosges Mountains

**DOI:** 10.1002/ece3.5668

**Published:** 2019-09-27

**Authors:** Olivier Gimenez, Sylvain Gatti, Christophe Duchamp, Estelle Germain, Alain Laurent, Fridolin Zimmermann, Eric Marboutin

**Affiliations:** ^1^ CEFE CNRS EPHE IRD Univ Montpellier Univ Paul Valéry Montpellier 3 Montpellier France; ^2^ Office National de la Chasse et de la Faune Sauvage Gières France; ^3^ Office National de la Chasse et de la Faune Sauvage Gap France; ^4^ Centre de Recherche et d'Observation sur les Carnivores (CROC) Lucy France; ^5^ KORA Muri Switzerland

**Keywords:** camera trapping, large carnivores, noninvasive sampling, photo identification, spatially explicit capture–recapture models

## Abstract

Obtaining estimates of animal population density is a key step in providing sound conservation and management strategies for wildlife. For many large carnivores however, estimating density is difficult because these species are elusive and wide‐ranging. Here, we focus on providing the first density estimates of the Eurasian lynx (*Lynx lynx*) in the French Jura and Vosges mountains. We sampled a total of 413 camera trapping sites (with two cameras per site) between January 2011 and April 2016 in seven study areas across seven counties of the French Jura and Vosges mountains. We obtained 592 lynx detections over 19,035 trap days in the Jura mountains and 0 detection over 6,804 trap days in the Vosges mountains. Based on coat patterns, we identified a total number of 92 unique individuals from photographs, including 16 females, 13 males, and 63 individuals of unknown sex. Using spatial capture–recapture (SCR) models, we estimated abundance in the study areas between 5 (*SE* = 0.1) and 29 (0.2) lynx and density between 0.24 (*SE* = 0.02) and 0.91 (*SE* = 0.03) lynx per 100 km^2^. We also provide a comparison with nonspatial density estimates and discuss the observed discrepancies. Our study is yet another example of the advantage of combining SCR methods and noninvasive sampling techniques to estimate density for elusive and wide‐ranging species, like large carnivores. While the estimated densities in the French Jura mountains are comparable to other lynx populations in Europe, the fact that we detected no lynx in the Vosges mountains is alarming. Connectivity should be encouraged between the French Jura mountains, the Vosges mountains, and the Palatinate Forest in Germany where a reintroduction program is currently ongoing. Our density estimates will help in setting a baseline conservation status for the lynx population in France.

## Graphical abstract

We provide the first density estimates for the Eurasian lynx (*Lynx lynx*) in the French Jura and Vosges mountains. Our study is yet another example of the advantage of combining spatial capture–recapture methods and noninvasive sampling techniques to estimate density for elusive and wide‐ranging species like large carnivores. While the estimated densities in the French Jura mountains are comparable to other lynx populations in Europe, the situation in the Vosges mountains is alarming. Our density estimates will help in setting a baseline conservation status for the lynx population in France.

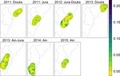

## 1 INTRODUCTION

Obtaining estimates of animal population density is a key step in providing sound conservation and management strategies for wildlife (Williams, Nichols, & Conroy, 2002). For many large carnivores however, estimating density is difficult because these species are elusive and wide‐ranging, resulting in low detection rates (Obbard, Howe, & Kyle, 2010). To deal with these issues, noninvasive techniques, such as camera trapping and DNA sampling, are increasingly used (Kelly, Betsch, Wultsch, Mesa, & Mills, 2012). These noninvasive techniques generate data that can be analyzed with capture–recapture methods to estimate densities (Royle, Chandler, Sollmann, & Gardner, 2014).

Standard capture–recapture models for closed populations (Otis, Burnham, White, & Anderson, 1978) have long been used to estimate animal abundance and density, including many large carnivores (Gerber, Ivan, & Burnham, 2014; Mumma, Zieminski, Fuller, Mahoney, & Waits, 2015). However, when converting abundance into density, density estimates are highly sensitive to the size of user‐defined area assumed to reflect the effective sampling area (White, Anderson, Burnham, & Otis, 1982). In addition, individual heterogeneity in detection due to spatial variation in the distance of home ranges to the sampling devices may lead to biased density estimates (Otis et al., 1978). Spatial capture–recapture (SCR) models deal with these issues by explicitly incorporating spatial locations of detections (Borchers, 2012; Borchers & Efford, 2008; Efford, 2004; Royle & Young, 2008), and they are increasingly used to estimate densities of large carnivores (Alexander, Gopalaswamy, Shi, & Face, 2015; Broekhuis & Gopalaswamy, 2016; Goldberg et al., 2015; López‐Bao et al., 2018; Pesenti & Zimmermann, 2013; Stetz, Mitchell, & Kendall, 2018).

Here, we focus on the threatened Eurasian lynx (*Lynx lynx*) in the French Jura and Vosges mountains (see Chapron et al., 2014 for a map of its distribution in Europe; see also https://www.lcie.org/Large-carnivores/Eurasian-lynx for recent updates). As in many regions of western Europe (Breitenmoser, 1998), lynx was extirpated from France between the 17th and 20th centuries due to habitat degradation, persecution by humans and decrease in prey availability (Vandel & Stahl, 2005). Shortly, after their initial reintroduction in Switzerland in the 1970s (Breitenmoser, Breitenmoser‐Würsten, & Capt, 1998), lynx naturally increased their range and started recolonizing France by repopulating forests on the French side of the Jura (Vandel & Stahl, 2005). Reintroductions also occurred in the French Vosges mountains between 1983 and 1993 with the perspective of establishing a population there (Vandel, Stahl, Herrenschmidt, & Marboutin, 2006). The species is listed as endangered in the IUCN Red list and is of conservation concern in France due to habitat fragmentation, poaching, and collisions with cars and trains. Currently, the French population of lynx is restricted to three mountain ranges: the Vosges in northeastern France, the Jura, and the Alps, with little connectivity between them most likely due to human‐made linear infrastructures. While the Northern Alps are slowly being recolonized with lynx mostly coming from the Jura (Marboutin et al., 2012), the Jura holds the bulk of the French lynx population. In contrast, the lynx presence in the Vosges mountains remained stable following the reintroductions and has been continuously decreasing since 2005 (Laurent et al., 2012).

Despite their conservation status, little information on abundance and density of lynx in France exist. In this study, we used SCR and standard capture–recapture models to provide the first estimate of lynx abundance and density using camera trap surveys implemented in the French Jura and Vosges mountains from 2011 to 2016. Based on these results, we discuss research and management priorities for the effective conservation of lynx in France.

## 2 METHODS

### 2.1 Ethics statement

We used noninvasive methods for data collection, which did not involve manipulation or handling of any living organism. Therefore, approval from an animal ethics committee was not required. Cameras were set on public or private forests with the permission of local authorities or local owners, respectively. We advertised the study and the presence of camera traps to the local stakeholders and the public visiting the areas. In agreement with French legislation, we deleted photographs permitting the identification of goods or people.

### 2.2 Study area and sampling design

**Figure 1:**
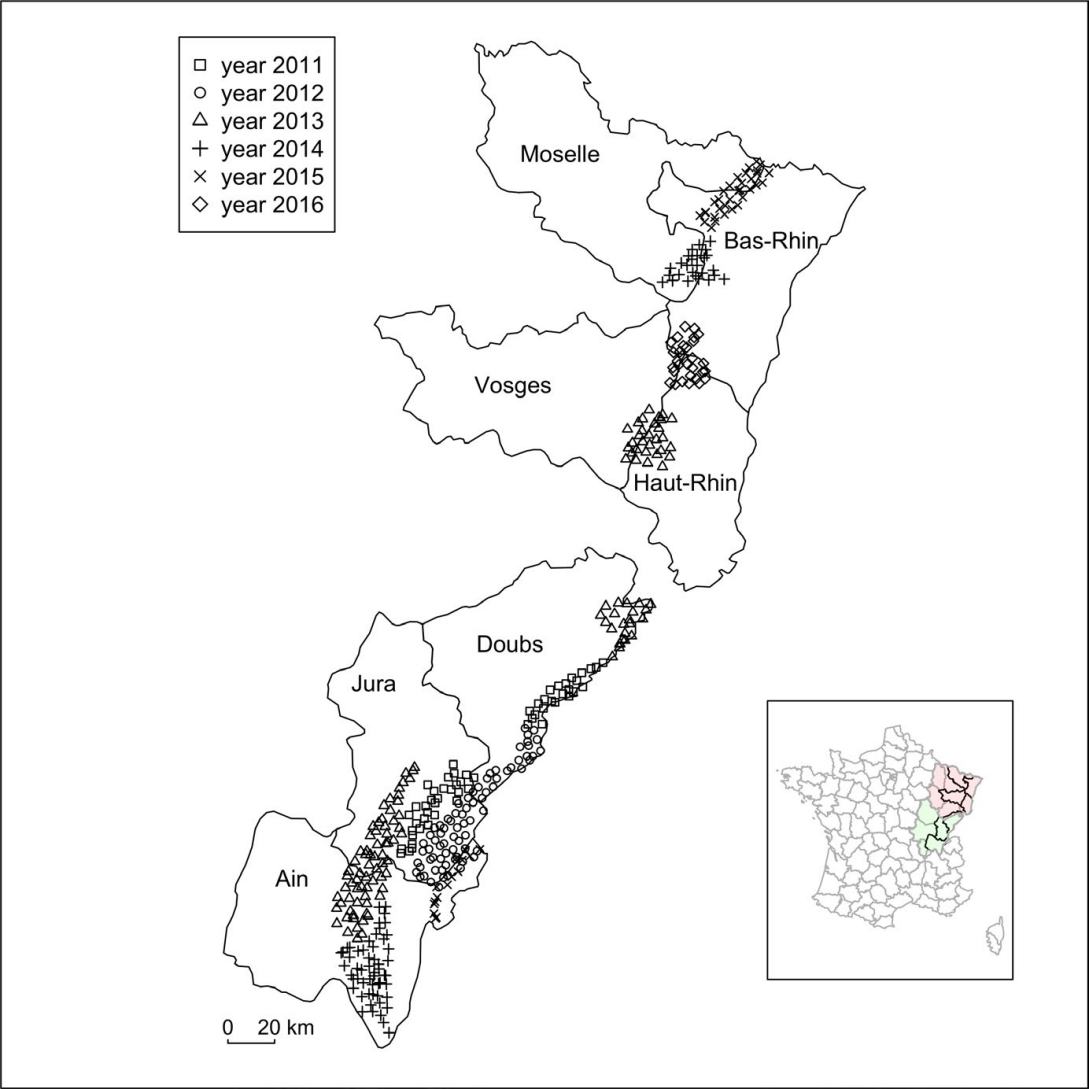
Map of the study area in the French Jura and Vosges mountains. The study area encompassed seven counties (Ain, Jura and Doubs in the Jura mountains and Vosges, Haut‐Rhin, Bas‐Rhin and Moselle in the Vosges mountains) that were monitored through 413 camera trapping sites (298 in the Jura mountains and 115 in the Vosges mountains; two camera traps were set per site), each within a 2.7 × 2.7 km cell. The inset map represents the French counties (gray borders), the counties that were considered in the study (black borders), the Jura mountains (green shaded area) and the Vosges mountains (red shaded area)

The study area encompassed three counties of the French Jura mountains, namely Ain, Doubs and Jura, and four counties of the Vosges mountains, namely Vosges, Haut‐Rhin, Bas‐Rhin, and Moselle (Figure 1). Elevation ranged from 163 to 1,718 m above sea level in the Jura mountains, and from 104 to 1,422 m in the Vosges mountains. The human population density was 88 per km^2^ in the Jura mountains and 170 per km^2^ in the Vosges mountains. The Jura mountains were 50% forest on average (Breitenmoser et al., 2007), and the Vosges mountains were 70% forest on average (DREAL Grand Est, 2018). The rest of the area was permanent pastures, arable land, and human settlements. Sampling occurred over 6 years, between January 2011 and April 2016, mostly in winter and spring, with surveys lasting between 2 and 4 months. We considered two study areas in 2011, 2014, and 2015, three study areas in 2013, and one study area in 2012 and 2016 through camera trapping (Figure 1).

We divided each study area into a grid of 2.7 × 2.7 km cells applying a systematic design where one out of two cells was sampled (Zimmermann, Breitenmoser‐Würsten, Molinari‐Jobin, & Breitenmoser, 2013), hence ensuring that at least one camera trap was set in each potential lynx home range (between 100 and 250 km^2^, see Breitenmoser‐Würsten et al., 2007). To maximize detectability, we set (nonbaited) camera traps in forested habitats, based on previous signs of lynx presence and on local knowledge, at optimal locations where landscape and terrain features were likely to channel lynx movements on more predictable paths (on forest roads, hiking trails, and to a lesser extent on game paths; Blanc, Marboutin, Gatti, & Gimenez, 2013). Camera was settled within a single session continuously during 60 days between February and beginning of March with little variation between sites.

At each trapping location, we set two Xenon white flash camera traps (models: Capture, Ambush and Attack; Cuddeback) with passive infrared trigger mechanisms to photograph both flanks of an animal. We checked camera traps weekly to change memory cards, batteries and to remove fresh snow after heavy snowfall. Based on unique coat patterns, we identified individual lynx on photographs (Zimmermann & Foresti, 2016). The recognition of individual was computer‐induced, not fully automated. We used the Extract‐compare^©^ software that compares the lynx spot pattern with a library of previously extracted pattern and proposes potential matches according to a score (http://conservationresearch.org.uk/Home/ExtractCompare). The observer can confirm the lynx identification or not and browse through the highest‐ranking proposed matches. The final decision is made by the observer based on an additional visual examination of the entire photograph set for this particular lynx. Pictures for which no match was found with the software were visually checked against our entire photograph library. Only when the match was undeniable was the individual recorded as a match, otherwise it was recorded as a new individual. All captures that did not fit automated or associated visual confirmation with no doubt, because of a poor picture quality (e.g., blurry, overexposed), were classified as “unconfirmed” and excluded from the analyses. We recorded the date, time, sex whenever possible, and location of each photographic capture of a lynx. During the time of year our study took place, juvenile lynx (<1 year old) can still be with their mother (Zimmermann, Breitenmoser‐Würsten, & Breitenmoser, 2005). In our analysis, we retained only independent lynx, that is, adult lynx or emancipated individuals based on physical characteristics or previous knowledge of their age or status (from photographic evidence). We defined a capture occasion as 5 successive trap nights (Blanc et al., 2013), dissociating trapping events from individual photograph to avoid pseudo‐replications.

### 2.3 Spatial capture–recapture analyses

We used spatial capture–recapture (SCR) models to estimate lynx densities (Royle et al., 2014). In contrast with standard (nonspatial) capture–recapture models, SCR models use the spatial locations of captures to infer the activity center (or home range) of each individual. We assumed that individual encounters are Bernoulli random variables with individual‐ and trap‐specific detection probabilities. More precisely, the detection probability *p_ij_* of an individual *i* at trap *j* is assumed to decrease as the distance (*d_ij_*) from its activity center increases according to a detection function. We used the half‐normal detection function, *p_ij_* = *p*_0_ exp(−$d_{\mathrm{ij}}^{2}$/(2*σ* ^2^)), where *p*_0_ is the probability of detecting an individual when the trap is located exactly at its center of activity and *σ* is the spatial scale (or movement) parameter that controls the shape of the detection function. For one of the two study areas in the French Jura mountains in years 2011 and 2013, we detected only a few individuals (see the columns Doubs in Table 1). To increase the effective sample size, we combined the data from these two sampling areas using common detection and spatial parameters for both areas, while estimating density separately (e.g., Rocha, Sollmann, Ramalho, Ilha, & Tan, 2016). We defined a state‐space, that is, the area encompassing all potential activity centers of the observed individuals, by building a grid that buffered outermost camera trap locations by 15 km (corresponding to at least 2*σ*; Royle et al., 2014) with a resolution of 1.5 km (or pixels of area 2.25 km^2^). We fitted SCR models in the maximum likelihood framework using the R package oSCR (Sutherland, Muñoz, Miller, & Grant, 2016; Sutherland, Royle, & Linden, 2019).

**Table 1:** Main characteristics and results of the lynx camera trap survey carried out in (a) the French Jura mountains and (b) the French Vosges mountains

(a) Year/County	2011/Doubs	2011/Jura	2012/Jura & Doubs	2013/Doubs	2013/Ain & Jura	2014/Ain	2015/Ain
Period of trap activity	January–April	February–April	February–April	February–April	February–April	February–April	February–May
Number of active camera traps	48	66	148	44	142	118	30
Number of trapping days (average/area)	63	59	69	63	58	59	99
Number of capture occasions^i^	15	15	17	14	13	13	21
Number of detections	22	42	130	25	117	158	38
Number of detected individuals	4	9	21	6	19	23	10
Number of females, unknown, males	1, 1, 2	1, 7, 1	2, 14, 5	1, 4, 1	2, 13, 4	4, 16, 3	2, 8, 0
Number of detections/ind: mean, min, max	3, 2, 4	2.8, 1, 6	2.5, 1, 10	2.7, 1, 6	3.6, 1, 11	3.3, 1, 9	2.2, 1, 5

(b) Year/County	2013/Haut‐Rhin & Vosges	2014/Bas‐Rhin & Moselle	2015/Bas‐Rhin & Moselle	2016/Bas & Haut‐Rhin
Period of trap activity	December–January	February–April	February–April	February–April
Number of active traps	60	50	60	60
Number of trapping days (average/area)	52	59	57	59
Number of detections	0	0	0	0

i A capture occasion is defined as 5 successive trap days.

For comparison, we also estimated abundance using standard (nonspatial) capture–recapture models (Otis et al., 1978). We dropped the spatial information and considered only the detections and nondetections for each individual. We considered two models, M0 in which the detection probability is the same for all individuals, and Mh in which the detection probability varies among individuals. We fitted standard models in the maximum likelihood framework using the R package Rcapture (Baillargeon & Rivest, 2007). We estimated density as the ratio of estimated abundance over an effective trapping area (ETA). ETA was estimated by adding a buffer to the trapping area equal to the mean maximum distance moved (MMDM) or half of it (HMMDM). We calculated the MMDM by averaging the maximum distances between capture locations for all individuals detected at more than one site.

## 3 RESULTS

We collected data from 413 camera trapping sites (two camera traps were set per site) resulting in 25,839 trap days (Table 1). In total, we identified 92 lynx over 532 detection events in the Jura mountains, including 16 females, 13 males, and 63 individuals of unknown sex. The number of detections per individual was 2.6 on average and varied from 1 to 11. In contrast, we collected no lynx photograph in the Vosges mountains; therefore, we did not proceed with analyses for this area.

For the Jura mountains, abundance estimates were similar whether we used spatial or nonspatial models, although always slightly higher for the former. Estimated abundance among study areas varied between 5 (*SE* = 0.1) and 29 (0.2) lynx in the spatial analyses, between 4 (0.7) and 23 (0.7) with model M0, and between 5 (1.7) and 28 (3.6) with model Mh. Estimated density varied between 0.24 (0.02) and 0.91 (0.03) lynx per 100 km^2^ in the spatial analyses (Table 2). In the nonspatial analyses, the density varied between 0.31 (0.05) and 0.78 (0.02) lynx per 100 km^2^ under model M0 and between 0.34 (0.06) and 0.95 (0.12) under model Mh when the MMDM was used. When we used HMMDM, the density varied between 0.57 (0.10) and 1.46 (0.16) lynx per 100 km^2^ under model M0 and between 0.67 (0.12) and 1.43 (0.16) under model Mh.

**Table 2:** Lynx abundance and density estimates obtained from spatial and nonspatial capture–recapture analyses of camera trapping data collected in the French Jura mountains

Year/County	2011/Doubs	2011/Jura	2012/Jura‐Doubs	2013/Doubs	2013/Ain‐Jura	2014‐Ain	2015‐Ain
SCR abundance (*SE*)	5 (0.1)	12 (0.1)	29 (0.2)	7 (0.1)	21 (0.1)	29 (0.2)	12 (0.1)
SCR density (*SE*)	0.24 (0.02)	0.44 (0.02)	0.67 (0.02)	0.36 (0.02)	0.54 (0.02)	0.91 (0.03)	0.64 (0.03)
*p*_0_ logit scale (*SE*)	−2.94 (0.24)		−2.01 (0.20)	−2.57 (0.20)		−2.34 (0.19)	−3.01 (0.42)
*σ* log scale (*SE*)	8.89 (0.14)		8.54 (0.08)	8.95 (0.06)		8.80 (0.07)	8.97 (0.19)
M0 abundance (*SE*)	4 (0.7)	9 (0.7)	21 (0.6)	6 (0.3)	19 (0.8)	23 (0.7)	11 (1.2)
Mh abundance (*SE*)	5 (1.7)	10 (1.8)	25 (2.8)	7 (1.2)	25 (4.1)	28 (3.6)	11 (1.2)
MMDM (km)	9.1	16.2	8.9	9.1	18.2	13.6	12.1
ETA with MMDM (km^2^)	1,991	2,930	3,089	1,171	4,954	2,936	1,549
M0 density MMDM (*SE*)	0.31 (0.05)	0.31 (0.02)	0.68 (0.02)	0.51 (0.02)	0.38 (0.02)	0.78 (0.02)	0.71 (0.08)
Mh density MMDM (*SE*)	0.39 (0.13)	0.34 (0.06)	0.81 (0.09)	0.60 (0.10)	0.50 (0.08)	0.95 (0.12)	0.70 (0.08)
ETA with HMMDM (km^2^)	697	1,491	2,111	659	2,673	1,668	753
M0 density HMMDM (*SE*)	0.57 (0.10)	0.60 (0.05)	0.99 (0.03)	0.91 (0.05)	0.71 (0.03)	1.38 (0.04)	1.46 (0.16)
Mh density HMMDM (*SE*)	0.72 (0.24)	0.67 (0.12)	1.18 (0.13)	1.06 (0.18)	0.93 (0.15)	1.68 (0.21)	1.43 (0.16)

Note

Densities are provided in number of lynx per 100 km^2^. For 2011 and 2013, parameters of the spatial capture–recapture model (*p*_0_ and *σ*) are common to both areas in each year.

Abbreviations: ETA, effective trapping area; HMMDM, half mean maximum distance moved; M0, the (nonspatial) capture–recapture model with homogeneous detection probability; Mh, the (nonspatial) capture–recapture model with heterogeneous detection probability; MMDM, mean maximum distance moved; SCR, spatial capture–recapture; *SE*, standard error.

**Figure 2:**
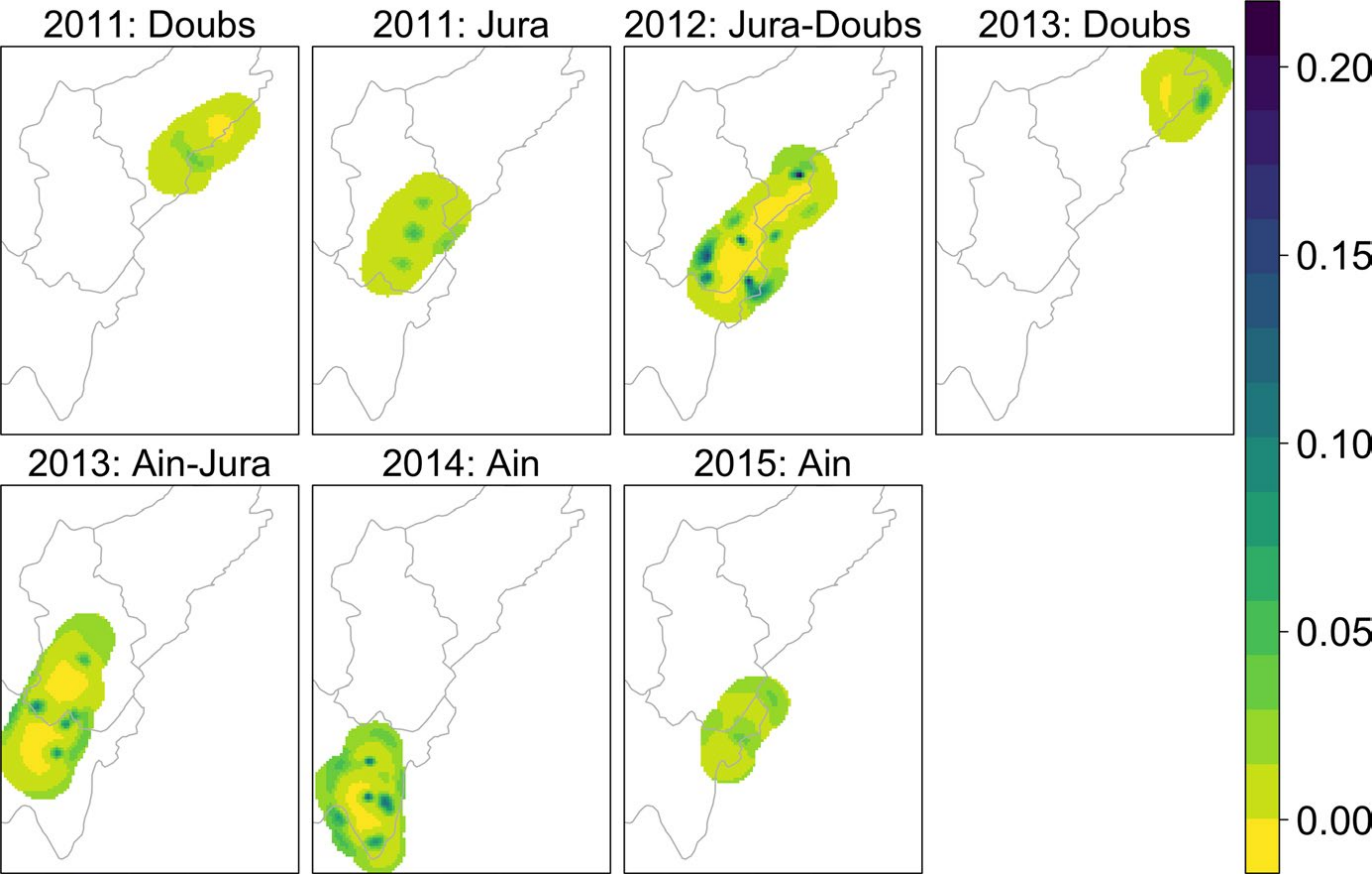
Lynx (*Lynx lynx*) density maps in the French Jura mountains. The density scale is in lynx per 2.25 km^2^ (pixel resolution is 1,500 m × 1,500 m). We obtained the estimated abundance in each map by summing up the densities in each pixel altogether. Yellow is for low densities, green for medium densities, and blue for high densities; the density scales are specific to each map. Note that the interpretation of these plots as density maps is subject to caution (see the vignette “secr‐densitysurface” of the SECR R package; Efford, 2019)

From the spatial analyses, we used the model estimates to produce density surfaces within the state‐space (Figure 2). The density per pixel of area 2.25 km^2^ ranged from 0 to 0.20 individuals in the Jura mountains.

## 4 DISCUSSION

By using camera trap sampling and SCR models, we provided the first multi‐site density estimates for lynx that will help in setting a baseline conservation status for the French lynx population. The multi‐site dimension of our study allows exploring variability in the density estimates across landscapes. Our study is yet another example of the potential of combining SCR methods and noninvasive sampling techniques to estimate abundance and density for elusive and wide‐ranging species, like large carnivores (Alexander et al., 2015; Broekhuis & Gopalaswamy, 2016; Goldberg et al., 2015; López‐Bao et al., 2018; Pesenti & Zimmermann, 2013; Stetz et al., 2018).

When examining densities across study areas in the French Jura mountains, we found spatial variation between the three counties, with Doubs area having the lowest densities, Ain the highest densities, and Jura intermediate densities. Our density estimates were of similar magnitude to other lynx populations in Europe: 1.47 and 1.38 lynx/100 km^2^ in the Northwestern Swiss Alps (Pesenti & Zimmermann, 2013), 0.58 (Štiavnica mountains) and 0.81 individuals/100 km^2^ (Velká Fatra National Park) in Slovakia (Kubala et al., 2017) and 0.9 individuals/100 km^2^ in the Bavarian Forest National Park in Germany (Weingarth et al., 2012).

While Kubala et al. (2017) and Pesenti and Zimmermann (2013) used SCR models, (Weingarth et al., 2012) used standard capture–recapture models with HMMDM to estimate densities, which makes them difficult to compare (Gerber, Karpanty, & Kelly, 2012). Indeed, in other carnivore studies, the use of HMMDM also produced similar density estimates to SCR models (Pesenti & Zimmermann, 2013), while in others, including ours, the SCR estimates were closer to the MMDM estimates (Obbard et al., 2010) or intermediate between the MMDM and HMMDM estimates (Reppucci, Gardner, & Lucherini, 2011). When looking at reference values for densities across the distribution range of the species, it may be biologically meaningful to use the MMDM density estimate as a reference as it covers the whole potential of animal movements. On the other hand, because SCR models make space explicit whereas standard model‐based densities are sensitive to the definition of the effective sampling area, we recommend the use of SCR models to estimate lynx densities.

Our lynx density estimates might suffer from potential sources of bias that need to be discussed. First, the period of sampling is important to account for when setting up camera trap surveys (Weingarth et al., 2015). We conducted our survey outside the dispersal period, during the lynx mating season (February–March mostly). We did so to avoid capturing transient individuals and to increase detectability because of high lynx activity and relatively reduced human activities (Zimmermann & Foresti, 2016). However, some individuals might have moved in and out of the study areas, especially males who cover greater distances during the mating season. Whereas the presence of nonresident individuals can affect the calculation of (H)MMDM, and in turn density estimated with standard capture–recapture models, SCR density estimates were found to be robust to the presence of transient individuals (Royle, Fuller, & Sutherland, 2016). Second, males have larger home ranges than females (Pesenti & Zimmermann, 2013), which leads to heterogeneity in the SCR model parameter estimates. Because there were too few males and females identified and lots of individuals with unknown sex, sex‐specific SCR analyses (Sollmann et al., 2011) produced unreliable abundance and density estimates (results not shown). If detection heterogeneity is ignored in capture–recapture models, abundance is underestimated (Cubaynes et al., 2010), therefore our density estimates are probably biased low and should be considered as a conservative metric. The determination of sex could be improved by (a) combining the photographic surveys with genetic surveys, (b) conducting deterministic surveys over several years (e.g., Pesenti & Zimmermann, 2013), (c) conducting an opportunistic camera trapping survey all over years and setting camera traps at fresh lynx kills, (d) setting infrared flash camera traps capable of taking burst of images in rapid sequence at marking sites regularly used by the lynx (e.g., Vogt, Zimmermann, Kölliker, & Breitenmoser, 2014).

Last, we did not detect any individuals in the Vosges mountains, even though the sampling effort was similar to that implemented in the Jura mountains (Table 1). Despite the release of 21 lynx between 1983 and 1993 (out of which only 10 survived; Vandel et al., 2006), connectivity with the Jura mountains was and still is difficult to emerge because of artificial habitat fragmentation (highways and high‐speed train railways). In recent years, very few signs of presence of lynx have been collected in the Vosges mountains through opportunistic monitoring (mostly direct observations, more rarely footprints or hairs). In 2018, the regular presence area of lynx was of 400 km^2^ with signs of presence both in the north, in the center, and south of the massif. Currently, only two males are identified with photographs. One came from the Palatinate Forest in Germany (from a reintroduction program) and installed his home range in the Hautes‐Vosges in 2017. The second one came from the Jura mountains from where he dispersed in 2015. There are also some punctual incursions of lynx from Palatinate forest in the north Vosges. Overall, our findings are likely to be representative of the current critical situation of the lynx in the Vosges mountains.

We envision several perspectives to our work. First, while density estimates are of primary interest for conservation, understanding the mechanisms underlying trends in abundance is required to make sound conservation decisions (Williams et al., 2002). SCR models have been extended to open populations (Gardner, Reppucci, Lucherini, & Royle, 2010) and can be used to estimate demographic parameters (survival, reproduction) of large carnivores (Whittington & Sawaya, 2015). Unfortunately, because of logistic constraints, we could not sample the same areas over several years, which precludes a standard application of these models. A solution may lie in the combination of the data we collected through systematic camera trap surveys with additional data in the SCR framework, such as occupancy data (Blanc, Marboutin, Gatti, Zimmermann, & Gimenez, 2014) or opportunistic camera trap data (Tenan, Pedrini, Bragalanti, Groff, & Sutherland, 2017). Second, in addition to traffic‐induced mortality and conflicts with human activities, the expansion of lynx populations is limited by habitat fragmentation (Kramer‐Schadt, Revilla, Wiegand, & Breitenmoser, 2004), hence the need to assess connectivity with other populations (Zimmermann & Breitenmoser, 2007). SCR models can be used to quantify landscape connectivity by replacing the Euclidean distance between camera traps and home range centers by the least‐cost path (Royle, Chandler, Gazenski, & Graves, 2013; Sutherland, Fuller, & Royle, 2015). For lynx, this will require setting up traps across a gradient of habitat types, not only forested habitats, so that resistance to movement can be estimated.

In conclusion, our lynx density estimates for the French Jura mountains complement nicely the estimates recently provided for the Northwestern Swiss Alps (Pesenti & Zimmermann, 2013). The use of camera trapping coupled with SCR models in both France and Switzerland was the result of a cooperation between the two countries with the perspective of a transboundary monitoring (Gervasi et al., 2016; Vitkalova et al., 2018). This approach would prove useful to accurately estimate densities in other areas where habitats and prey availability might differ, and overall lynx detectability varies. Also, collecting and adding movement data from GPS‐collared lynx would be useful (Linden, Sirén, & Pekins, 2018; Tenan et al., 2017) to try and infer the connections between subpopulations.

The case can be made for monitoring the return of the lynx in the French Alps. Indeed, small‐scale camera trapping surveys and opportunistic observations are currently active and producing signs of lynx presence. However, the lack of a coordinated and intensive sampling effort prevents us from being able to estimate abundance and density and inferring trends.

In contrast, the situation in the Vosges mountains is alarming with no individuals detected over the study period. Because the Vosges mountains are located between the French Jura mountains and the Palatinate Forest in Germany where a reintroduction program is ongoing (program LIFE13 NAT/DE/000755), the lynx colonization in the Vosges mountains remains possible both by the north and the south. Incidentally, two cases of lynx dispersal in the Vosges mountains from neighboring mountains have been recently observed (Hurstel & Laurent, 2016; program LIFE13 NAT/DE/000755). To ensure the detection of lynx in the Vosges mountains, we recommend reinforcing collaborative monitoring by involving all field stakeholders and enhancing communication on the species signs of presence.

In this context, obtaining accurate and comparable lynx densities will be crucial to closely monitor population trends at the national scale and inform management policies for the effective conservation of the Eurasian lynx in France.

## CONFLICT OF INTEREST

None declared.

## AUTHOR CONTRIBUTIONS

OG wrote the paper and all co‐authors commented on the manuscript. OG and SG analyzed the data. AL, CD, EG, EM, and SG coordinated the study designs, the data collection, and interpretation, with help from FZ for setting the experimental design in the Jura mountains.

## Data Availability

The Eurasian lynx is an endangered species with high conservation stakes. Interactions with human activities are problematic and lead to poaching and anthropogenic pressures. Providing accurate information on lynx locations can be detrimental to the conservation status of the species. As a consequence, the original data could not be shared.
